# Phosphate solubilizing microorganisms as a driving force to assist mine phytoremediation

**DOI:** 10.3389/fbioe.2023.1201067

**Published:** 2023-05-11

**Authors:** Fei Chen, Jinyi Ma, Qiangliang Yuan, Zihua Yu

**Affiliations:** ^1^ Nanjing Museum Administration, Nanjing, China; ^2^ School of Horticulture, Nanjing Agricultural University, Nanjing, China; ^3^ Nanjing Museum, Nanjing, China

**Keywords:** phosphate solubilizing microorganisms, mine remediation, soil nutrients, plant growth, promotion and synergy

## 1 Phosphate solubilizing microorganisms (PSMs) are partners in promoting plant growth

PSMs are a group of beneficial microorganisms that mediate soil phosphorus cycling and biological utilization. The primary function of these micoorganisms is to release phosphorus from insoluble phosphorus compounds to promote the utilization of P by plants (Chen et al., 2019). PSMs category is very wide, including fungi, bacteria, arbuscular mycorrhizal fungi, actinomycetes, vesicular arbuscular mycorrhizae, etc. Among them, Penicillium and Aspergillus are representatives of fungi, while Enterobacter, Serratia and Pseudomonas are representatives of bacteria (Zaidi et al., 2016; Rawat et al., 2021). In the solubilization mechanism of soil phosphorus by PSM, mineralization of organic phosphorus by biological enzymes (Phytases) (Yadav and Tarafdar, 2011) and solubilization of inorganic phosphorus by organic acids are now widely accepted mechanisms (Chen et al., 2023). Furthermore, some PSMs can also release phosphorus sources from the environment by secreting inorganic acids, such as HCl and H_2_SO_4_ (Etesami et al., 2021; Rawat et al., 2021). While PSMs enhance plant growth primarily by providing soluble phosphorus to plants, they also improve the overall performance of plants by providing a variety of other growth-promoting substances. On the one hand, microorganisms produce antibiotics, hydrogen cyanide, iron carriers, antifungal compounds (such as PAL, phenolic compounds and flavonoids) and other biocontrol agents to enhance plant resistance to diseases and pests, and indirectly promote plant growth (Alori et al., 2017). On the other hand, phytohormones secreted by PSM, such as Indole-3-Acetic Acid (IAA), cytokinin, and gibberellin acid, also play an important role in regulating plant growth. Among them, IAA is the plant growth regulator most related to the physiological activity of plants, which has been widely studied. It promotes plants’ ability to absorb nutrients by stimulating root growth, expanding root surface area, and changing plant root morphology (Etesami et al., 2021). The promotion of plant growth by PSMs makes inoculation with PSMs a widely accepted and environmentally friendly method for improving agricultural productivity. Additionaly, the soil phosphorus dissolved by the PSMs often exceeds the demand of plants (Richardson and Simpson, 2011; Raymond et al., 2021), which allows potential excess phosphorus to be used in other environments. This is particularly relevant for ecosystems that rely on plant assistance for their composition, such as for seedling planting, environmental restoration, and landscape renovation. In these cases, PSMs hold significant promise for playing a crucial role in the future.

## 2 Phytoremediation is a low-cost and sustainable technology for mine remediation

Mining is a temporary industrial activity, which leads to drastic changes in the natural environment, such as altering the landscape and terrain, destroying vegetation, eliminating soil microorganisms and animals, producing a large amount of mine waste, and endangering both the aquifer and water source supply area (de Moura et al., 2022). In most cases, mining activities affect the resilience of the ecosystem and it is almost impossible to regenerate through natural regeneration. Therefore, it is necessary to use restoration techniques to restore ecosystems and protect biodiversity (Menegaki and Kaliampakos, 2012). Studies have shown that bioremediation techniques using plants and/or microorganisms are cheaper and more environmentally friendly than conventional physical and chemical techniques (Gong et al., 2018). However, one of the main challenges in the rehabilitation of mine sites is the re-establishment of self-sustaining vegetation. Due to the lack of vegetation, the erosion in these areas is intensified, resulting in the pollution of the surrounding areas. In addition, groundwater may be contaminated by leaching. The spread of this contamination may pose a threat to human health if it enters the food chain through drinking water and crops. However, due to the serious degradation of the mining area, the establishment and development of future plants are hindered and recovery is affected.

It is well known that soil degradation caused by mining activities mainly includes: 1) dissolution of heavy metals in waste forms high-concentration pollution stress; 2) Lack of organic matter and sandy structure and poor water storage capacity; 3) Loss of large amounts of nutrients such as N, P, and K; and 4) The functional homogeneity and poor diversity of microbial community of the native soil (Wong, 2003; Sheoran et al., 2011; Lebrun et al., 2021). Among them, heavy metals have negative effects on plant cell growth, root development, and photosynthesis by inhibiting enzyme function, disrupting the nucleic acid structure, and interfering with plant nutrient absorption (Etesami, 2018). In addition, phosphorus can form complexes with metal ions in the soil, making it mostly unavailable for plants to absorb (Rawat et al., 2021). These factors combine to make phytoremediation a difficult process to achieve in abandoned mines. Therefore, reducing heavy metal stress and regulating soil properties are the basis for improving phytoremediation in mining areas. In addition to the introduction of heavy metal resistant plant species or breeding mechanisms to improve the heavy metal tolerance of traditional plant species, the addition of soil amendments (e.g., mycorrhizal agents, biochar, phosphate minerals, etc.) can also help to mitigate the negative effects.

## 3 PSMs as potential regulators in mine remediation

The specific nature of pollution in mining sites often makes it difficult to achieve phytoremediation alone. Currently, most reclamation and remediation strategies used in abandoned mine sites are based on soil modification with chemicals, combined with plants for long-term remediation. However, these measures are often unsustainable because they tend to ignore the balance and enrichment effects of plant-associated microbes in the mine site. Microbial activities in abandoned mine sites are mainly in the topsoil (20–30 cm), water, tailings and sediments, rhizosphere environment of plants, and phyllosphere environment of plants (Thavamani et al., 2017). Although some of the native microorganisms in the mine site are able to evolve new resistance mechanisms to cope with the pressure of toxic pollutants, the abundance and diversity of microbial communities are bound to be greatly reduced, especially the microbial flora associated with plant growth promotion. This phenomenon also makes it difficult for new plants to settle in the environment. Therefore, in order to develop sustainable remediation methods, more attention should be paid to understanding and utilizing the role of plant-microorganism interactions in mine sites.

**FIGURE 1:**
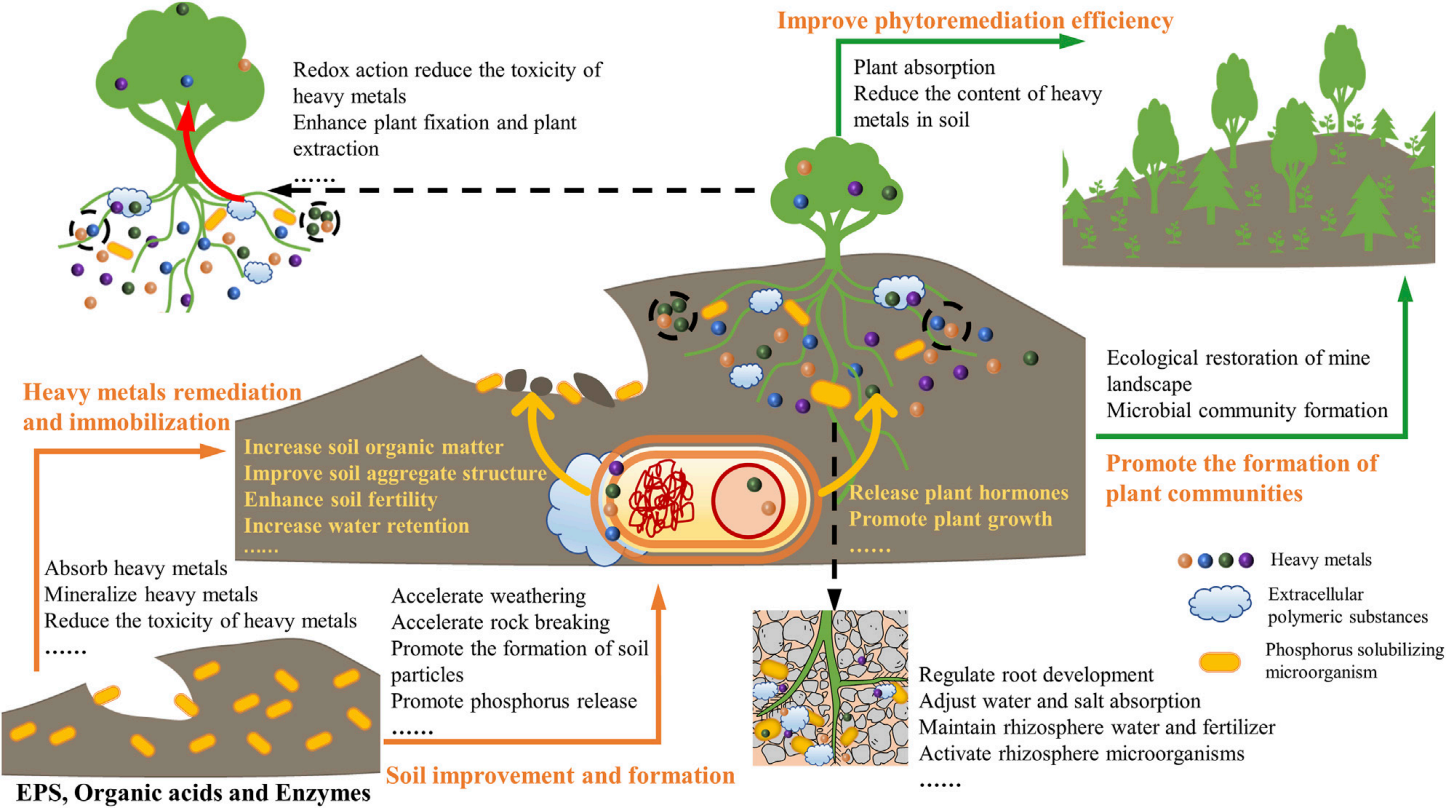
Schematic diagram of the mechanism of phosphate solubilizing microorganism assisted mine landscape restoration.

PSMs, which possesses both heavy metal remediation and plant growth promotion functions, may be an extremely promising option. The main advantage of PSMs is their better rhizosphere ability, such as the ability to colonize, grow, and develop faster in rhizosphere soil. The process of PSMs promoting plant remediation of mine pollution is mainly divided into two aspects: soil improvement and formation, and plant growth and protection (Figure 1).

(1) Soil improvement and formation: PSMs accelerate the weathering and decomposition of soil layers, as well as the formation of natural soil, and creates an environment for plants to take root (Tian et al., 2021; Yi et al., 2021). At the same time, functional microorganisms can change soil structure and nutrient cycling, increasing soil aggregate stability (Bruneel et al., 2019; da Silva et al., 2022; Thavamani et al., 2017).
(2) Plant growth and protection: Firstly, PSMs play an important role in promoting plant growth. It promotes nutrient circulation, improves the plant defenses against disease and pests, and increases plant growth by secreting plant auxin, dissolving phosphorus, fixing nitrogen, and other mechanisms. Secondly, the efficient immobilization function of PSMs for heavy metals is key to their assistance in mine phytoremediation. The phosphate released by PSMs can solidify and stabilize the free heavy metal ions (Lai et al., 2022). Furthermore, PSMs can absorb, accumulate, and complex heavy metal ions through cells (internal and surface) and by the secretion of organic substances (extracellular polymer, glutathione, etc.). Meanwhile, PSMs reduce the toxicity of heavy metals through its own redox, methylation, and other metabolic effects. In addition, PSMs directly promote the phytoremediation of heavy metals by improving the transport ability of metal ions in the rhizosphere (phytoextraction) or reducing the metal migration ability (phytostabilization) (Ahemad, 2015; Gupta and Kumar 2017). The two key mechanisms of PSMs make them an efficient growth promoter and an immobilizer of heavy metals in mine sites. In conclusion, in the joint remediation system of PSMs and plants, on the one hand, plants can immobilize heavy metals in soil or themselves, while PSMs can transform heavy metals into a less potentially toxic, lower bioavailable, and/or less mobile form than the initial form. At the same time, PSMs interact with plants to improve their growth by releasing P, plant auxin, pest inhibitors, etc., making the phytoremediation process more effective. On the other hand, vegetation coverage stabilize the soil, reduce erosion and leaching, and enrich the soil structure, thereby providing a cyclic gain effect for the expansion and reproduction of PSMs.

## 4 Prospect

PSMs play an important role in the establishment and development of plants and ecosystem functions in mine remediation. The use of PSMs in a degraded environment with high heavy metal pollution can become the driving force for the successful restoration of the transboundary ecosystem. In addition, PSMs have great potential to inoculate microorganisms into mine soil to improve plant growth and reduce metal toxicity. Although PSMs have not been widely promoted for use in mine remediation at present, the prospects for their application are self-evident. Therefore, the engineering application of PSMs in actual mines should be encouraged and promoted in future research. More attention should be paid to explore whether complex and rarely heavy metal species will have more severe stress effects and differences in remediation mechanisms for PSMs in special mining environments, as well as developing a combined remediation technology of PSMs with physical and chemical remediation techniques for mines.
