# Corrigendum: Phosphate solubilizing microorganisms as a driving force to assist mine phytoremediation

**DOI:** 10.3389/fbioe.2023.1273399

**Published:** 2023-10-02

**Authors:** Fei Chen, Jinyi Ma, Qiangliang Yuan, Zihua Yu

**Affiliations:** ^1^ Nanjing Museum Administration, Nanjing, China; ^2^ School of Horticulture, Nanjing Agricultural University, Nanjing, China; ^3^ Nanjing Museum, Nanjing, China

**Keywords:** phosphate solubilizing microorganisms, mine remediation, soil nutrients, plant Growth, promotion and synergy

In the published article, there was an error in **Section 1** Phosphate solubilizing microorganisms (PSMs) are partners in promoting plant growth **(**line 6 of the first paragraph) “Among them, *Penicillium, Aspergillus*, and *Bacillus* are representatives of fungi,” the genus *Bacillus* belongs to bacteria and not fungi.

This should have been written as “Among them, *Penicillium* and *Aspergillus* are representatives of fungi, while *Enterobacter*, *Serratia* and *Pseudomonas* are representatives of bacteria (Zaidi et al., 2016; Rawat et al., 2021).”

The authors apologize for this error and state that this does not change the scientific conclusions of the article in any way. The original article has been updated.

